# 0738. Mortality is associated with early tachycardia and cardiac troponin release in a fluid-resuscitated rat model of sepsis

**DOI:** 10.1186/2197-425X-2-S1-P60

**Published:** 2014-09-26

**Authors:** W Khaliq, M Singer

**Affiliations:** Bloomsbury Institute of Intensive Care Medicine, University College London, London, UK

## Introduction

Tachycardia and high troponin levels prognosticate for poor outcomes in human sepsis [1]. Reducing cardiac stress with beta-blockade has been proposed as an important therapeutic strategy as high catecholamine levels are injurious [2]. We have characterized a 72h fluid-resuscitated rat model of faecal peritonitis where prognostication can be made with high sensitivity and specificity at 6h from heart rate and stroke volume [3].

## Objectives

To determine whether non-survival is associated with early changes in troponin release and circulating catecholamine levels.

## Methods

Male Wistar rats (325±15g) underwent insertion of tunneled carotid arterial and jugular venous lines under isoflurane anaesthesia, followed by immediate i.p. injection of 4µl/g faecal slurry. Control animals were treated identically but without i.p. injection of slurry. Once awake, attachment to a swivel-tether system allowed animals to move freely and access food and water *ad libitum*. Fluid resuscitation (50:50 mixture of 5% dextrose/Hartmann's; 10ml/kg/h) was commenced at 2h. At 6h, echocardiography was used to measure heart rate and stroke volume. Animals were observed until 72h to assess survival. In a second experiment septic animals underwent echocardiography at 6h followed by sacrifice and blood and tissue sampling. We here report plasma catecholamine and troponin T levels (measured by ELISA) in predicted survivors and non-survivors, and sham-operated controls.

## Results

Septic animals (n=16) had a mortality rate of 56%, with death occurring between 18-36h. A heart rate cut point of 460/min measured at 6h prognosticated 3-day survival with sensitivity of 0.88 and specificity of 0.92. Clinical features of illness at this timepoint were however mild. Table 1 shows significant differences in haemodynamics and troponin levels between predicted survivors and non-survivors. Catecholamine levels, while elevated over non-septic controls, were similar.

**Table 1:** Table 1

	Control (n=6)	Predicted survival (n=6)	Predicted non-survival (n=6)
**Heart rate (bpm)**	390 ± 21	442 ± 17	488 ± 18*
**Stroke volume (mL)**	0.40 ± 0.03	0.25 ± 0.02	0.18 ± 0.02*
**Adrenaline (ng/mL)**	8.56 ± 0.42	9.44 ± 0.23	10.3 ± 0.18
**Noradrenaline (ng/mL)**	1.60 ± 0.25	3.21 ± 0.23	2.98 ± 0.27
**Troponin (pg/mL)**	171 ± 24	168 ± 15	311 ± 47*

Data shown as median ± SE; * p<0.05 ANOVA

## Conclusions

An association was seen between eventual non-survival and tachycardia, low stroke volume and myocardial injury (denoted by high troponin) at 6h after induction of sepsis. The impact of modulating cardiac stress on outcome merits further study.
